# Higher dose versus lower dose of antiviral therapy in the treatment of herpes zoster infection in the elderly: a matched retrospective population-based cohort study

**DOI:** 10.1186/2050-6511-15-48

**Published:** 2014-09-04

**Authors:** Ngan N Lam, Jamie L Fleet, Eric McArthur, Peter G Blake, Amit X Garg

**Affiliations:** 1 Department of Medicine, Division of Nephrology, Western University, London, ON N6A 3 K7, Canada; 2 Department of Epidemiology and Biostatistics, Western University, London, ON N6A 3 K7, Canada; 3 Institute for Clinical Evaluative Sciences (ICES), London, ON N6A 5 W9, Canada; 4 Kidney Clinical Research Unit, Room ELL-111, London Health Sciences Centre, 800 Commissioners Road East, London, ON N6A 4G5, Canada

**Keywords:** Administrative database, Epidemiology, Mortality, Neurotoxicity

## Abstract

**Background:**

Higher versus lower doses of antiviral drugs used to treat herpes zoster infection may lead to more adverse drug events in older adults, particularly those with chronic kidney disease.

**Methods:**

We conducted a matched retrospective population-based cohort study of older adults (mean 77 years) in Ontario, Canada who initiated in the outpatient setting a higher (n = 23,256) or lower (n = 3,876) dose of one of three oral antivirals for the treatment of herpes zoster between 2002 and 2011. The primary outcome was hospitalization within 30 days with evidence of a computed tomography (CT) scan of the head (a proxy for acute neurotoxicity). The secondary outcome was 30-day all-cause mortality.

**Results:**

A higher compared to lower dose of antiviral drug was not associated with an increased risk of hospitalization with an urgent CT scan of the head (247 [1.06%] events with higher dose versus 43 [1.11%] events with lower dose, relative risk 0.96, 95% confidence interval 0.69 to 1.33, p-value 0.79) and was not associated with a higher risk of all-cause mortality (63 [0.27%] events versus 15 [0.39%] events, relative risk 0.70, 95% confidence interval 0.40 to 1.23, p-value 0.21). Results were consistent in all subgroups, including those with and without chronic kidney disease.

**Conclusions:**

Initiating a higher compared to a lower dose of an antiviral drug for the treatment of herpes zoster was not associated with an increased risk of adverse drug events. The findings support the safety of these drugs in older adults as currently prescribed in routine care.

## Background

Acyclovir, valacyclovir (a pro-drug which is metabolized to acyclovir), and famciclovir are prescribed for the treatment of herpes zoster infection [1,2]. These drugs are commonly prescribed to older adults who are at risk of dose-related adverse drug reactions, particularly neurotoxicity with delirium [3,4]. In older patients with chronic kidney disease, the recommendation is to reduce the dose of these drugs to prevent systemic accumulation from reduced elimination (Table 1) [5-12]. There have been many case reports and case series of reversible acute neurological symptoms, such as delirium, resulting in hospitalization soon after the initiation of acyclovir, valacyclovir, or famciclovir [13-20]. Whether preferential use of a low dose of the antiviral drug minimizes this risk is unknown. Therefore, we conducted this study of older patients with herpes zoster infection to investigate whether initiation of a higher rather than lower dose of an oral antiviral drug in the outpatient setting is associated with more adverse drug events (neurotoxicity, death) within 30 days of prescription. We also considered whether any association between dose and adverse events differed in the presence of chronic kidney disease.

**Table 1:** Oral antiviral dosing for acute herpes zoster in popular drug prescribing references

	**Higher dose (mg/day)**^**a**^	**Lower dose (mg/day)**^**a**^	**UpToDate recommendations (dose in mg/day) **[7]**-**[9]	**Compendium of pharmaceuticals and specialties (dose in mg/day) **[10]**-**[12]
Acyclovir	4,000	3,200	4,000	4,000
		2,400	CrCl 10–25 mL/min/1.73 m^2^: 2,400	CrCl 10–25 mL/min/1.73 m^2^: 2,400
		1,600	CrCl <10 mL/min/1.73 m^2^: 1,600	CrCl <10 mL/min/1.73 m^2^: 1,600
		800		
Valacyclovir	3,000	2,000	3,000	3,000
		1,500	CrCl 15–30 mL/min: 2,000	CrCl 15–30 mL/min: 2,000
		1,000	CrCl <15 mL/min: 1,000	CrCl <15 mL/min: 1,000
		500		
Famciclovir	1,500	1,000	1,500	1,500
		500	CrCl 40–59 mL/min: 1,000	CrCl 40–59 mL/min/1.73 m^2^: 1,000
		350^b^	CrCl 20–39 mL/min: 500	CrCl 20–39 mL/min/1.73 m^2^: 500
			CrCl <20 mL/min: 250	CrCl <20 mL/min/1.73 m^2^: 250

^a^Dose categories as defined in this study include 50 mg dose above or below the cut-points.

^b^A dose of 350 mg/day was used instead of 250 mg/day because it was more commonly dispensed in our region with <20 patients being prescribed a dose of <300 mg/day.

*Abbreviation: CrCl* Creatinine Clearance.

## Methods

### Design and setting

We conducted this study at the Institute for Clinical Evaluative Sciences (ICES) according to a pre-specified protocol that was approved by the research ethics board at Sunnybrook Health Sciences Centre (Toronto, Canada). Participant informed consent was not required for this study. We conducted a retrospective, population-based, matched cohort study of older adults using linked healthcare databases in Ontario, Canada. Ontario has approximately 13 million residents, 2 million of whom are aged 65 years or older [21]. Residents have universal access to hospital care and physician services and those aged 65 or older have universal prescription drug coverage. The reporting of this study follows guidelines set out for observational studies (Additional file 1: Table S1) [22].

### Data sources

We ascertained patient characteristics, drug use, covariate information, and outcome data using records from six databases. We obtained vital statistics from the Registered Persons Database (RPDB), which contains demographic information on all Ontario residents ever issued a health card. We used the Ontario Drug Benefit (ODB) database to identify prescription drug use, including dispensing date, quantity of pills, dose, and number of days supplied. This database contains highly accurate records of all outpatient prescriptions dispensed to patients aged 65 years or older, with an error rate of less than 1% [23]. We identified diagnostic and procedural information on all hospitalizations and emergency room visits from the Canadian Institute for Health Information Discharge Abstract Database (CIHI-DAD) and the National Ambulatory Care Reporting System (NACRS), respectively. We obtained covariate information from the Ontario Health Insurance Plan (OHIP) database, which includes health claims for inpatient and outpatient physician services. We used the ICES Physician Database (IPDB) to ascertain antiviral drug prescriber information. Previously, we have used these databases to research health adverse drug events and health outcomes, including acyclovir-induced acute kidney injury [24-26]. With the exception of antiviral prescriber specialty and income quintile (missing in 14.5% and 0.4% of patients, respectively), the databases were complete for all variables used in this study. Given the ability of our databases to capture healthcare activity province-wide, the only loss to follow-up would be if patients emigrated from Ontario (a rate estimated to be less than 1% per year) [27]. The database codes used in the analysis are defined in Additional file 1: Table S2.

### Patients

We established a cohort of residents aged ≥66 years in Ontario, Canada who filled a new outpatient prescription with ≥7-day supply for oral acyclovir, valacyclovir, or famciclovir from April 2002 to December 2011, a period spanning 9 years. We restricted our analysis to those who had evidence of a herpes zoster diagnosis in the 90 days prior to or 30 days following the time of the prescription (database diagnosis codes presented in Additional file 1: Table S2). The date of the first eligible prescription for a study antiviral served as the index date for that patient and marked the start date of follow-up. We excluded the following patients from the analysis: i) those in their first year of eligibility for prescription drug coverage (age 65) to avoid incomplete medication records, ii) those living in long-term care facilities since residents may have frequent episodes of confusion or delirium for many reasons, iii) those with end-stage renal disease, iv) those who had a prescription for any antiviral in the prior 180 days in order to capture new usage, v) those who had a prescription for more than one type of antiviral on the index date in order to compare mutually exclusive groups, and vi) those who had a hospital admission or discharge on their index date or a hospital discharge in the prior two days to ensure these were new outpatient antiviral prescriptions (as patients continuing an antiviral treatment initiated in hospital would have their outpatient antiviral prescription dispensed on the same or next day of hospital discharge).

To select two groups of antiviral users that were well-balanced on the baseline characteristics we measured in this study, we matched each low-dose user with a high-dose user on a 1:6 basis using the following variables: age (within two years), sex, presence of chronic kidney disease, and type of antiviral prescribed (acyclovir, valacyclovir, or famciclovir). In Ontario, the validated algorithm for chronic kidney disease identifies older adults with a median estimated glomerular filtration rate (eGFR) of 38 mL/min per 1.73 m^2^ (interquartile range 27 to 52), whereas its absence identifies those with a median eGFR of 69 mL/min per 1.73 m^2^ (interquartile range 56 to 82) [28].

### Antiviral dose

To align with recommendations in drug prescribing references, a higher dose of antiviral therapy was defined as at least 4,000 mg/day for acyclovir, 3,000 mg/day for valacyclovir, and 1,500 mg/day for famciclovir. A lower dose of antiviral therapy was defined as 3,200 mg/day, 2,400 mg/day, 1,600 mg/day or 800 mg/day for acyclovir, 2,000 mg/day, 1,500 mg/day, 1,000 mg/day, or 500 mg/day for valacyclovir, and 1,000 mg/day, 500 mg/day, or 350 mg/day for famciclovir (Table 1).

### Outcomes

We followed all patients for 30 days after the index date for the assessment of two pre-specified outcomes. The primary outcome was hospital admission with evidence of an urgent computed tomography (CT) scan of the head within the first five days of admission (inclusive of any scans performed in the emergency room preceding an admission). Based on prospective studies of common clinical practice, neuroimaging is frequently used in the routine evaluation of patients who present to hospital acutely confused, even among those without focal neurological findings or head trauma [29-31]. Unlike diagnostic codes for acute delirium, the receipt of a CT scan of the head is well coded in our data sources (these codes have high sensitivity and specificity for receipt of the imaging as they are associated with physician reimbursement) [32]. We also expected urgent CT scans of the head conducted for reasons unrelated to antiviral dosing to occur at a similar frequency in higher and lower dose groups; therefore, not impacting estimates of difference in risk. We have used this outcome of urgent CT scans of the head in other population-based drug safety studies to characterize the risk of drug-induced delirium [33]. Our secondary outcome was all-cause mortality. Death is accurately coded in our data sources (sensitivity 94%, positive predictive value 100%) [34].

### Statistical analysis

We compared baseline characteristics between those prescribed a higher or lower antiviral dose using standardized differences [35]. This metric describes differences between group means relative to the pooled standard deviation and is considered a meaningful difference if greater than 10%. We estimated the odds ratio and 95% confidence intervals for inpatient CT scan of the head with higher antiviral dose compared to lower antiviral dose using conditional logistic regression analyses (accounting for matched sets). We interpreted odds ratios as relative risks which was appropriate given the low incidence of observed events. We examined the relative risk between higher dose and lower dose (referent dose) antiviral and each outcome first in the entire matched cohort and then in four pre-defined subgroups based on: age, sex, presence of chronic kidney disease, and antiviral type. We examined whether relative risks differed among subgroups using tests for interaction. We conducted all analysis with Statistical Analysis Software (SAS) version 9.2 (SAS Institute Incorporated, Cary, North Carolina, USA, 2008).

## Results

We identified 77,381 eligible older adults who were prescribed outpatient oral antiviral drug for the treatment of herpes zoster infection (higher dose, n = 73,383 versus lower dose, n = 3,998). After the match, there were a total of 27,132 eligible patients of which 23,256 (85.7%) received a higher antiviral dose and 3,876 (14.3%) received lower antiviral dose (referent dose). A diagram of the cohort selection is represented in Additional file 1: Figure S1. The baseline characteristics of patients before and after the match are presented in Table 2. After the match, the baseline characteristics of the two dose groups were nearly identical (all standardized differences for 17 measured variables between the groups were ≤8%). The mean age was 77 years (standard deviation 7.1 years) and 63% were women. Three-quarters of the prescriptions were written by primary care physicians with 67% of prescriptions written for valacyclovir. Ophthalmologist prescribed <1% of the antivirals and <0.3% of patients had a diagnosis for herpes zoster involving the eye.

**Table 2:** Baseline characteristics

	**Unmatched**			**Matched**		
	**Higher dose**^**a **^**(n = 73,383)**	**Lower dose**^**b **^**(n = 3,998)**	**Standardized difference**^**c**^	**Higher dose**^**a **^**(n = 23,256)**	**Lower dose**^**b **^**(n = 3,876)**	**Standardized difference**^**c**^
Demographics						
Age, years	75.9 [6.8]	77.0 [7.3]	0.15	76.7 [7.1]	76.7 [7.1]	0
Women	45,613 (62.2)	2,541 (63.6)	0.03	14,682 (63.1)	2,447 (63.1)	0
Year of cohort entry						
2002 - 2003	11,318 (15.4)	514 (12.9)	0.07	3,083 (13.3)	505 (13.0)	0.01
2004 - 2005	14,016 (19.1)	595 (14.9)	0.11	4,096 (17.6)	572 (14.8)	0.08
2006 - 2007	14,930 (20.3)	807 (20.2)	0	4,641 (20.0)	779 (20.1)	0
2008 - 2009	15,783 (21.5)	993 (24.8)	0.08	5,320 (22.9)	964 (24.9)	0.05
2010 - 2011	17,336 (23.6)	1,089 (27.2)	0.08	6,116 (26.3)	1,056 (27.2)	0.02
Income quintile^d^						
First (lowest)	13,937 (19.0)	831 (20.8)	0.04	4,404 (18.9)	804 (20.7)	0.05
Second	15,526 (21.2)	802 (20.1)	0.03	4,886 (21.0)	777 (20.1)	0.02
Third (middle)	14,231 (19.4)	842 (21.1)	0.04	4,469 (19.2)	818 (21.1)	0.05
Fourth	14,521 (19.8)	751 (18.8)	0.03	4,620 (19.9)	730 (18.8)	0.03
Fifth (highest)	14,976 (20.4)	755 (18.9)	0.04	4,808 (20.7)	731 (18.9)	0.05
Missing	192 (0.26)	17 (0.43)	0.03	69 (0.3)	16 (0.4)	0.02
Rural location^e^	10,864 (14.8)	505 (12.6)	0.06	3,027 (13.0)	491 (12.7)	0.01
Modified Charlson score^f^						
0	53,431 (72.8)	2,731 (68.3)	0.10	16,623 (71.5)	2,684 (69.3)	0.05
1	8,024 (10.9)	445 (11.1)	0.01	2,560 (11.0)	428 (11.0)	0
2	6,350 (8.7)	400 (10.0)	0.05	2,086 (9.0)	383 (9.9)	0.03
≥3	5,578 (7.6)	422 (10.6)	0.10	1,987 (8.5)	381 (9.8)	0.04
Co-morbidities^g^						
CKD^h^	3,799 (5.2)	425 (10.6)	0.20	1,896 (8.2)	316 (8.2)	0
Chronic liver disease	2,420 (3.3)	138 (3.5)	0.01	735 (3.2)	135 (3.5)	0.02
COPD	2,994 (4.1)	212 (5.3)	0.06	1,010 (4.3)	198 (5.1)	0.04
CAD^i^	25,840 (35.2)	1,548 (38.7)	0.07	8,427 (36.2)	1,478 (38.1)	0.04
Diabetes mellitus^j^	11,016 (15.0)	699 (17.5)	0.07	3,636 (15.6)	671 (17.3)	0.05
Heart failure	8,469 (11.5)	611 (15.3)	0.11	2,985 (12.8)	571 (14.7)	0.06
Stroke/TIA	1,556 (2.1)	109 (2.7)	0.04	513 (2.2)	99 (2.6)	0.02
Herpes zoster (eye)	89 (0.12)	8 (0.20)	0.02	30 (0.13)	8 (0.21)	0.02
Antiviral type						
Acyclovir	4,095 (5.6)	297 (7.4)	0.08	1,692 (7.3)	282 (7.3)	0
Valacyclovir	29,482 (40.2)	2,694 (67.4)	0.57	15,528 (66.8)	2,588 (66.8)	0
Famciclovir	39,806 (54.2)	1,007 (25.2)	0.62	6,036 (26.0)	1,006 (26.0)	0
Medications^k^						
Anticonvulsants	3,079 (4.2)	178 (4.5)	0.01	988 (4.3)	167 (4.3)	0
Gabapentin	420 (0.57)	24 (0.60)	0	137 (0.59)	22 (0.57)	0
Antidepressants	11,201 (15.3)	663 (16.6)	0.04	3,756 (16.2)	636 (16.4)	0.01
Antipsychotics	1,431 (2.0)	88 (2.2)	0.02	503 (2.2)	86 (2.2)	0
Barbituates	115 (0.16)	7 (0.18)	0	45 (0.19)	7 (0.18)	0
Benzodiazepines	13,794 (18.8)	809 (20.2)	0.04	4,446 (19.1)	774 (20.0)	0.02
Histamine2-receptor antagonists	6,191 (8.4)	337 (8.4)	0	1,911 (8.2)	325 (8.4)	0.01
Dopamine agonists	326 (0.44)	22 (0.55)	0.02	124 (0.53)	22 (0.57)	0
Muscle relaxants	399 (0.54)	25 (0.63)	0.01	132 (0.57)	25 (0.64)	0.01
Opioids	15,738 (21.5)	957 (23.9)	0.06	5,076 (21.8)	923 (23.8)	0.05
Overactive bladder medications	2,169 (3.0)	149 (3.7)	0.04	704 (3.0)	142 (3.7)	0.04
Prescribing physician						
General practitioner	55,723 (75.9)	3,015 (75.4)	0.01	17,671 (76.0)	2,925 (75.5)	0.01
Ophthalmologist	272 (0.37)	28 (0.70)	0.05	90 (0.39)	27 (0.70)	0.04
Neurologist^l^	41 (0.06)	≤5 ( − )	( − )	12 (0.05)	≤5 ( − )	( − )
Other	7,538 (10.3)	372 (9.3)	0.03	2,350 (10.1)	360 (9.3)	0.03
Missing	9,809 (13.4)	581 (14.5)	0.03	3,133 (13.5)	562 (14.5)	0.03

Data presented as number (percent) except for age which is presented as mean [standard deviation].

*Abbreviations: CAD* Coronary Artery Disease, *CKD* Chronic Kidney Disease, *COPD* Chronic Obstructive Pulmonary Disease, *TIA* Transient Ischemic Attack.

^a^Higher dose of antiviral defined as 4,000 mg/day for acyclovir, 3,000 mg/day for valacyclovir, and 1,500 mg/day for famciclovir.

^b^A lower dose of antiviral defined as 3,200 mg/day, 2,400 mg/day, 1,600 mg/day or 800 mg/day for acyclovir, 2,000 mg/day, 1,500 mg/day, 1,000 mg/day, or 500 mg/day for valacyclovir, and 1,000 mg/day, 500 mg/day, or 350 mg/day for famciclovir.

^c^Standardized differences are less sensitive to sample size than traditional hypothesis tests. They provide a measure of the difference between groups divided by the pooled standardized difference; a value >10% (0.1) is interpreted as a meaningful difference between the groups [35].

^d^Income was categorized into quintiles based on average neighbourhood income on the index date.

^e^Rural location indicates a population <10,000.

^f^Assessed with an algorithm using diagnosis codes from hospitalizations in the five years prior; patients with no hospitalizations during this period were given a value of zero.

^g^Co-morbid diagnoses were ascertained from administrative database codes in the five years preceding the index date.

^h^Identified individuals with chronic kidney disease using an algorithm of diagnosis codes validated in our region for older adults [28]. The algorithm identified patients with a median estimated glomerular filtration rate (eGFR) of 38 mL/min per 1.73 m^2^ (interquartile range 27 to 52), whereas its absence identified patients with a median eGFR of 69 mL/min per 1.73 m^2^ (interquartile range 56 to 82).

^i^Coronary artery disease includes receipt of coronary artery bypass graft surgery, percutaneous coronary intervention, and diagnoses of angina.

^j^Identified individuals with diabetes through medication use including oral hypoglycemic and insulin.

^k^Medication use was assessed in the 180 days prior to the index date.

^l^Due to privacy issues, values ≤5 are suppressed.

**Figure 1:**
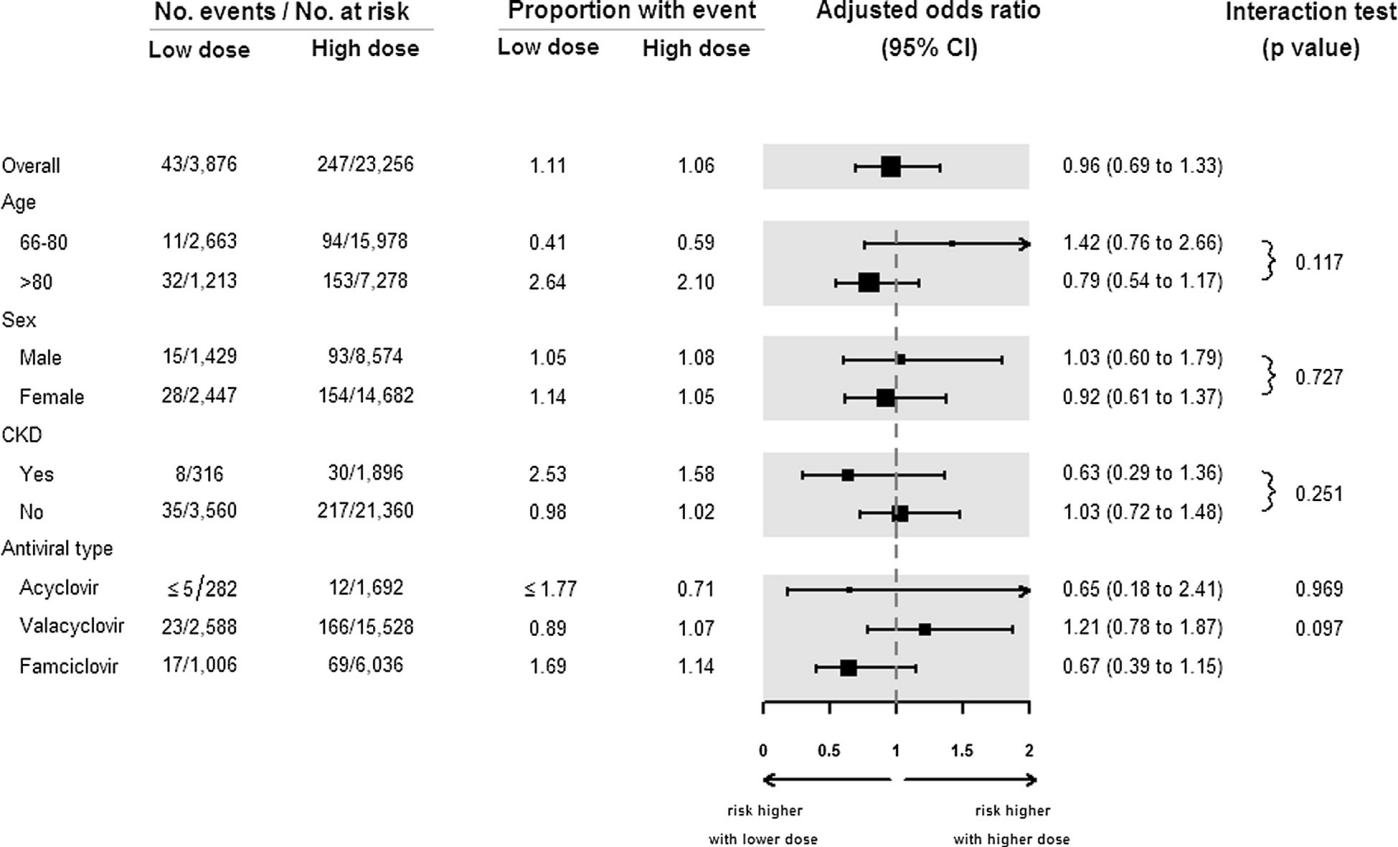
Subgroup analysis of the risk of hospital admission with urgent CT scan of the head.

Compared to lower antiviral dose, the initiation of higher antiviral dose was not associated with a higher risk of hospitalization with urgent CT scan of the head (247 [1.06%] events with higher dose versus 43 [1.11%] events with lower dose, relative risk 0.96, 95% confidence interval 0.69 to 1.33, p-value 0.79) (Table 3). Figure 1 presents the association between antiviral dose and the primary outcome in four pre-defined subgroups: by age, sex, presence of chronic kidney disease, and antiviral type. There was no association between antiviral dose and hospital admission with urgent CT scan of the head in any of the subgroups, including those with and without chronic kidney disease (p-value for interaction 0.25). There was also no difference between the two dose groups in the incidence of all-cause mortality (63 [0.27%] events with higher dose versus 15 [0.39%] events with lower dose, relative risk 0.70, 95% confidence interval 0.40 to 1.23, p-value 0.21) (Table 3).

**Table 3:** 30-day outcomes of hospital admission with urgent CT scan of the head and all-cause mortality

	**Number of events (Percent)**		**Relative risk**	**P-value**
	**Higher dose**^**a **^**(n = 23,256)**	**Lower dose**^**b **^**(Referent) (n = 3,876)**	**[95% confidence interval]**	
Hospital admission with urgent CT scan of the head	247 (1.06%)	43 (1.11%)	0.96 [0.69 to 1.33]	0.79
All-cause mortality	63 (0.27%)	15 (0.39%)	0.70 [0.40 to 1.23]	0.21

Abbreviations: CT, Computed Tomography.

^a^Higher dose of antiviral defined as 4,000 mg/day for acyclovir, 3,000 mg/day for valacyclovir, and 1,500 mg/day for famciclovir.

^b^A lower dose of antiviral defined as 3,200 mg/day, 2,400 mg/day, 1,600 mg/day or 800 mg/day for acyclovir, 2,000 mg/day, 1,500 mg/day, 1,000 mg/day, or 500 mg/day for valacyclovir, and 1,000 mg/day, 500 mg/day, or 350 mg/day for famciclovir.

## Discussion

In this population-based study of over 27,000 older patients, we found no association between initiating oral antiviral treatment for herpes zoster at a higher versus lower dose and the risk of hospital admission within 30 days with evidence of an urgent CT scan of the head. A similar association was observed in patients with and without chronic kidney disease, although given the smaller number of patients with chronic kidney disease, the estimates were less precise with wider confidence intervals. The 30-day risk of mortality was also no different between the two dose groups.

Acyclovir, valacyclovir, and famciclovir are commonly prescribed antivirals used in routine outpatient care for the treatment of herpes zoster infection in the elderly. In randomized controlled trials, these drugs are similarly effective in reducing the duration and severity of painful lesions, preventing complications, and decreasing the frequency of recurrence [1,2]. A known adverse event of these oral antiviral drugs is neurotoxicity [13-20]. The symptoms of drug-induced neurotoxicity include tremor, confusion, hallucinations, and coma and can often be difficult to distinguish from herpes encephalitis. With these antivirals, symptoms typically occur within days of drug initiation and generally resolve with drug discontinuation. In one case series of 35 patients with neuropsychiatric symptoms during acyclovir treatment, risk factors included older age, the presence of chronic kidney disease, and co-administration of other potentially neurotoxic medications [13].

Given the results of these prior pharmacokinetic studies and case reports, we were concerned we would observe an increased risk of major adverse events when a higher rather than lower dose of an antiviral drug was initiated in older adults, particularly those with concomitant chronic kidney disease. The findings from this study are reassuring and support the safety of these drugs in older adults as currently prescribed in routine care.

Our study has a number of strengths. To our knowledge, it is the first population-based assessment of adverse outcomes from higher dose versus lower dose antiviral treatment. The study was made possible by our province’s universal health care benefits, which provides information on all health care encounters for all Ontarians including accurate records of outpatient prescriptions to older patients. Consequently, there were a large number of patients who received antiviral prescriptions and this provided good precision for the estimates obtained for the primary outcome (there were over 200 events and the 95% confidence interval for the point estimate of the primary outcome was 0.69 to 1.33; confidently ruling out a 1.4 or higher risk).

Our study does have some limitations. For reasons of feasibility, in this retrospective study, we relied on urgent neuroimaging within the available data sources as a proxy for the diagnosis of acute altered mental status. A preferred methodology would be a prospective study with independent blinded outcome adjudication and detailed serial measures of cognitive function. In such an effort, kidney function values could also be recorded, rather than a validated algorithm for chronic kidney disease as used in our study. We were encouraged by the marked similarities in the baseline characteristics after matching and in the consistency of the observed association among various subgroups. However, in our study the antiviral dose was not randomly assigned, and with all observational studies, we may have failed to account for important unknown or unmeasured confounding variables. Our cohort consisted of patients over the age of 65 years. It is reassuring that 99% of elderly patients in routine care did not present to hospital with urgent CT scan of the head after use of either dose of oral antivirals. While we did not study younger patients in the present study, their incidence of adverse events would be expected to be even less.

## Conclusions

In this study, initiating a higher compared to a lower dose of an antiviral drug for the treatment of herpes zoster was not associated with an increased risk of adverse drug events. The findings support the safety of these drugs in older adults as currently prescribed in routine care.

## Abbreviations

CIHI-DAD: Canadian Institute for Health Information Discharge Abstract Database; CT: Computed tomography; eGFR: Estimated Glomerular Filtration Rate; ICES: Institute for Clinical Evaluative Sciences; IPDB: ICES Physician Database; NACRS: National Ambulatory Care Reporting System; ODB: Ontario Drug Benefit; OHIP: Ontario Health Insurance Plan; RPDB: Registered Persons Database; SAS: Statistical Analysis Software.

## Competing interests

The authors declare that they have no competing interests.

## Authors’ contributions

NNL, EM, and AXR conceived of the study, participated in its design, and drafted the manuscript. EM performed the statistical analysis. All authors read and approved the final manuscript.

## Pre-publication history

The pre-publication history for this paper can be accessed here:

http://www.biomedcentral.com/2050-6511/15/48/prepub

## Supplementary Material

Additional file 1: Table S1 STROBE checklist. **Table S2.** Databases and coding definitions for baseline characteristics and study outcomes. **Figure S1.** Cohort selection. Click here for file
