# Supplementary material for: Higher dose versus lower dose of antiviral therapy in the treatment of herpes zoster infection in the elderly: a matched retrospective population-based cohort study
Source: BMC Pharmacol Toxicol. 2014 Sep 4;15:48. doi: 10.1186/2050-6511-15-48 (PMC4158397; doi:10.1186/2050-6511-15-48)

Supplementary Table 1. STROBE checklist

STROBE checklist			
	Item No	Recommendation	Reported on Page #
**Title and abstract**	1	(a) Indicate the study's design with a commonly used term in the title or the abstract	Abstract
		(b) Provide in the abstract an informative and balanced summary of what was done and what was found	Abstract
**Introduction**			
Background/rationale	2	Explain the scientific background and rationale for the investigation being reported	Introduction
Objectives	3	State specific objectives, including any prespecified hypotheses	Introduction
**Methods**			
Study design	4	Present key elements of study design early in the paper	Methods
Setting	5	Describe the setting, locations, and relevant dates, including periods of recruitment, exposure, follow-up, and data collection	Methods
Participants	6	(a) Give the eligibility criteria, and the sources and methods of selection of participants. Describe methods of follow-up	Methods
		(b) For matched studies, give matching criteria and	Methods

		number of exposed and unexposed	
Variables	7	Clearly define all outcomes, exposures, predictors, potential confounders, and effect modifiers. Give diagnostic criteria, if applicable	Methods Supplementary Table 2
Data sources/ measurement	8	For each variable of interest, give sources of data and details of methods of assessment (measurement). Describe comparability of assessment methods if there is more than one group	Methods
Bias	9	Describe any efforts to address potential sources of bias	Discussion
Study size	10	Explain how the study size was arrived at	Methods
Quantitative variables	11	Explain how quantitative variables were handled in the analyses. If applicable, describe which groupings were chosen and why	Methods
Statistical methods	12	(a) Describe all statistical methods, including those used to control for confounding	Methods
		(b) Describe any methods used to examine subgroups and interactions	Methods
		(c) Explain how missing data were addressed	Methods
		(d) If applicable, explain how loss to follow-up was addressed	Not Applicable
		(e) Describe any sensitivity analyses	Methods Results
**Results**			

Participants	13	(a) Report numbers of individuals at each stage of study—e.g. numbers potentially eligible, examined for eligibility, confirmed eligible, included in the study, completing follow-up, and analysed	Supplementary Figure 1
		(b) Give reasons for non-participation at each stage	Supplementary Figure 1
		(c) Consider use of a flow diagram	Supplementary Figure 1
Descriptive data	14	(a) Give characteristics of study participants (e.g. demographic, clinical, social) and information on exposures and potential confounders	Table 2
		(b) Indicate number of participants with missing data for each variable of interest	Table 2
		(c) Summarise follow-up time (e.g. average and total amount)	Results
Outcome data	15	Report numbers of outcome events or summary measures over time	Table 3
Main results	16	(a) Give unadjusted estimates and, if applicable, confounder-adjusted estimates and their precision (e.g. 95% confidence interval). Make clear which confounders were adjusted for and why they were included	Table 3
		(b) Report category boundaries when continuous variables were categorized	Table 2
		(c) If relevant, consider translating estimates of	Not

		relative risk into absolute risk for a meaningful time period	Applicable
Other analyses	17	Report other analyses done—e.g. analyses of subgroups and interactions, and sensitivity analyses	Results Figure 1
**Discussion**			
Key results	18	Summarise key results with reference to study objectives	Discussion
Limitations	19	Discuss limitations of the study, taking into account sources of potential bias or imprecision. Discuss both direction and magnitude of any potential bias	Discussion
Interpretation	20	Give a cautious overall interpretation of results considering objectives, limitations, multiplicity of analyses, results from similar studies, and other relevant evidence	Discussion
Generalisability	21	Discuss the generalisability (external validity) of the study results	Discussion
**Other information**			
Funding	22	Give the source of funding and the role of the funders for the present study and, if applicable, for the original study on which the present article is based	Disclosure

Supplementary Table 2. Databases and coding definitions for baseline characteristics and study outcomes

Characteristic/Condition	Database	Codes
Age, Sex, Income, Rural	RPDB	
Year of Cohort Entry	ODB	
Prescribing Physician	IPDB	
Modified Charlson Score	CIHI-DAD	
Chronic Kidney Disease	CIHI-DAD	ICD-9: 250.40, 403.0, 403.1, 403.9, 404.0, 404.1, 404.9, 585, 586, 588.8, 588.9
		ICD-10: E10.2, E11.2, E13.2, E14.2, I12, I13, N08, N18, N19
	OHIP	OHIP: 403, 585
Chronic Liver Disease	CIHI-DAD	ICD-9: 070, 275.0, 275.1, 456.1, 456.2, 571, 572.2, 572.3, 572.4, 572.8, 573, 782.4, 789.1, 789.5, V026
		ICD-10: B16, B17, B18, B19, B94.2, E83.0, E83.1, I85, K70, K71.3, K71.4, K71.5, K71.7, K72.1, K72.9, K73, K74, K75.3, K75.4, K75.8, K75.9, K76, K77, R16.0, R16.2, R17, R18, Z22.5
	OHIP	OHIP: 070, 571, 573, Z551, Z554
Chronic Obstructive Pulmonary Disease	CIHI-DAD	ICD-9: 491, 492, 496
		ICD10: J41, J43, J44
Coronary Artery Disease	CIHI-DAD	ICD-9: 410, 412, 413, 414, 429.2, 429.5, 429.6, 429.7
		ICD-10: I20, I21, I22, I23, I24, I25, R93.1, T82.2, Z95.5, Z95.8, Z95.9
		CCI: 1.IJ.26, 1.IJ.27, 1.IJ.50 1.IJ.54, 1.IJ.57, 1.IJ.76
		CCP: 48.01, 48.02, 48.03, 48.04, 48.05, 48.1, 48.2, 48.3
	OHIP	OHIP: E646, E651, E652, E654, E655, G262, G298, R741, R742,

		R743, Z434, Z448, 410, 412, 413
Diabetes mellitus	ODB[a]	
Heart Failure	CIHI-DAD	ICD-9: 425, 428, 514, 518.4<br>ICD-10: I25.5, I50.0, I50.1, I50.9, J81<br>CCI: 1.HP.53, 1.HP.55, 1.HZ.53.GRFR, 1.HZ.53.LAFR, 1.HZ.53.SYFR<br>CCP: 49.61, 49.62, 49.63, 49.64
	OHIP	OHIP: R701, R702, Z429, 428
Stroke/TIA	CIHI-DAD	ICD-9: 431, 434, 435.8, 435.9, 436<br>ICD-10: G45, H34.1, I61, I62.9, I63.0, I63.1, I63.2, I63.3, I63.4, I63.5, I63.8, I63.9, I64
Herpes zoster (nervous system)	CIHI-DAD	ICD-9: 053.1<br>ICD-10: B02.2
Herpes zoster (eye)	CIHI-DAD	ICD-9: 053.2<br>ICD-10: B02.3
Herpes zoster (other)	CIHI-DAD	ICD-9: 053.7, 053.8, 053.9<br>ICD-10: B02.7, B02.8, B02.9
	OHIP	OHIP: 053
CT scan of the head	CIHI-DAD	CCI: 3AN20, 3EA20, 3ER20
	OHIP	OHIP: X188, X400, X401, X402, X405, X408
Mortality	RPDB	Vital status field

[a] Patients with diabetes were identified through the use of prescription oral hypoglycemic agents or insulin

Abbreviations: CCI, Canadian Classification of Health Interventions; CCP, Canadian Classification of Diagnostic, Therapeutic and Surgical Procedures; CIHI-DAD, Canadian Institute for Health Information

hospital Discharge Abstract Database; CT, Computed Tomography; ICD-9 International Classification of Disease, Ninth Revision; ICD-10, International Classification of Disease, Tenth Revision; IPDB, ICES (Institute for Clinical Evaluative Sciences) Physician Database; ODB, Ontario Drug Benefit; OHIP, Ontario Health Insurance Plan; RPDB, Registered Persons Database of Ontario; TIA, Transient Ischemic Attack.

Supplementary Figure 1. Cohort selection

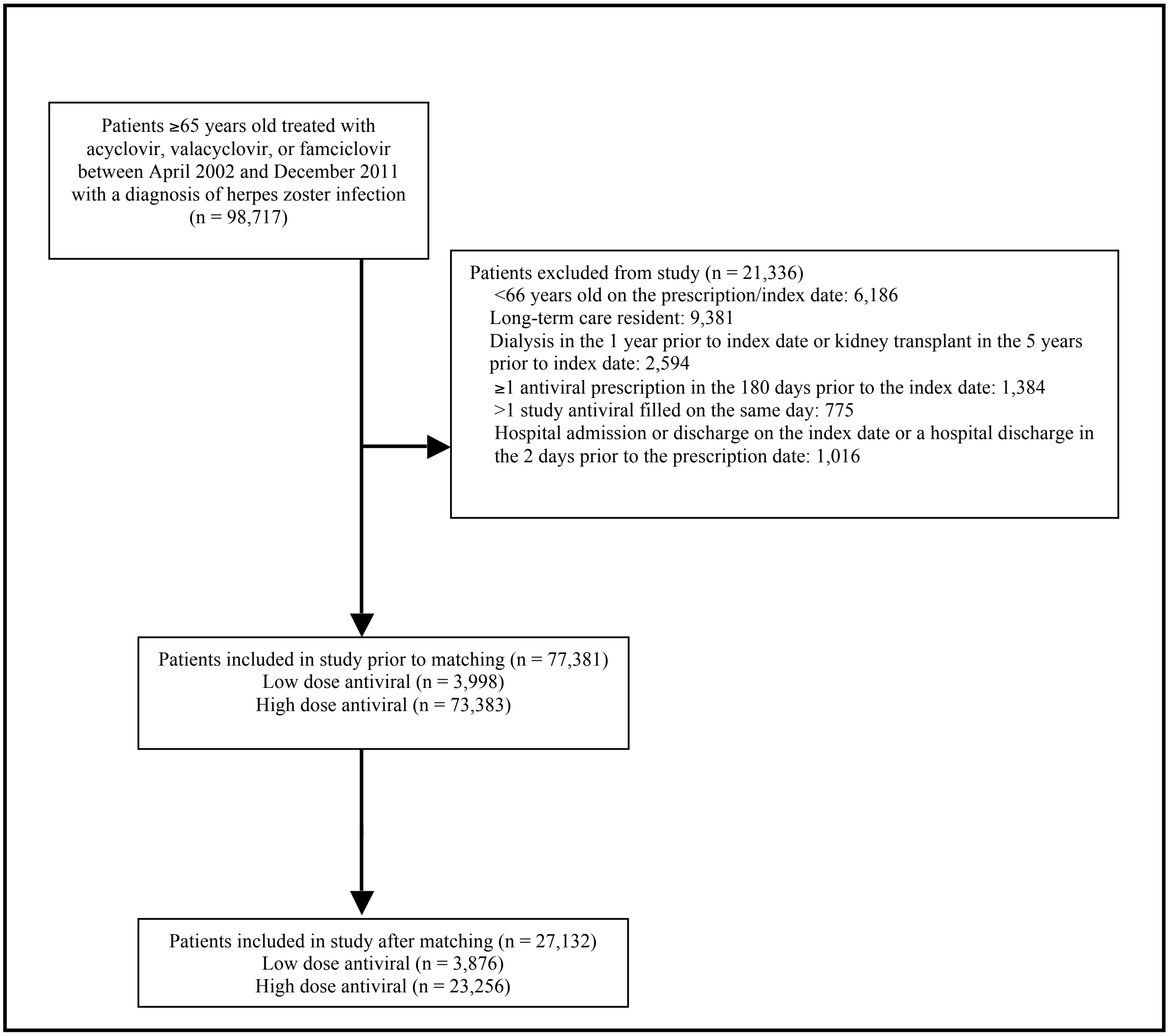

Supplement: Additional file 1: Table S1 — STROBE checklist. Table S2. Databases and coding definitions for baseline characteristics and study outcomes. Figure S1. Cohort selection. [file 2050-6511-15-48-S1.pdf]
